# Behavioral and Histopathological Assessment of Adult Ischemic Rat Brains after Intracerebral Transplantation of NSI-566RSC Cell Lines

**DOI:** 10.1371/journal.pone.0091408

**Published:** 2014-03-10

**Authors:** Naoki Tajiri, David M. Quach, Yuji Kaneko, Stephanie Wu, David Lee, Tina Lam, Ken L. Hayama, Thomas G. Hazel, Karl Johe, Michael C. Wu, Cesar V. Borlongan

**Affiliations:** 1 Center of Excellence for Aging & Brain Repair, Department of Neurosurgery and Brain Repair, University of South Florida College of Medicine, Tampa, Florida, United States of America; 2 Neuralstem, Inc., Rockville, Maryland, United States of America; 3 Neurodigitech, LLC., San Diego, California, United States of America; National Institutes of Health, United States of America

## Abstract

Stroke is a major cause of death and disability, with very limited treatment option. Cell-based therapies have emerged as potential treatments for stroke. Indeed, studies have shown that transplantation of neural stem cells (NSCs) exerts functional benefits in stroke models. However, graft survival and integration with the host remain pressing concerns with cell-based treatments. The current study set out to investigate those very issues using a human NSC line, NSI-566RSC, in a rat model of ischemic stroke induced by transient occlusion of the middle cerebral artery. Seven days after stroke surgery, those animals that showed significant motor and neurological impairments were randomly assigned to receive NSI-566RSC intracerebral transplants at two sites within the striatum at three different doses: group A (0 cells/µl), group B (5,000 cells/µl), group C (10,000 cells/µl), and group D (20,000 cells/µl). Weekly behavioral tests, starting at seven days and continued up to 8 weeks after transplantation, revealed dose-dependent recovery from both motor and neurological deficits in transplanted stroke animals. Eight weeks after cell transplantation, immunohistochemical investigations via hematoxylin and eosin staining revealed infarct size was similar across all groups. To identify the cell graft, and estimate volume, immunohistochemistry was performed using two human-specific antibodies: one to detect all human nuclei (HuNu), and another to detect human neuron-specific enolase (hNSE). Surviving cell grafts were confirmed in 10/10 animals of group B, 9/10 group C, and 9/10 in group D. hNSE and HuNu staining revealed similar graft volume estimates in transplanted stroke animals. hNSE-immunoreactive fibers were also present within the corpus callosum, coursing in parallel with host tracts, suggesting a propensity to follow established neuroanatomical features. Despite absence of reduction in infarct volume, NSI-566RSC transplantation produced behavioral improvements possibly via robust engraftment and neuronal differentiation, supporting the use of this NSC line for stroke therapy.

## Introduction

Stroke is a major unmet clinical need with only one current FDA-approved drug, the tissue plasminogen activator (tPA) [1]–[5]. The efficacy of tPA is limited to 4.5 hours after stroke onset and benefits only about 3% of ischemic stroke patients [6]–[8]. The advent of stem cell therapy opens the possibility of regenerating the injured brain and may prove effective in stroke beyond the acute phase of the disease [9]–[13]. With the increasing diversity of stem cell sources emerging for donor cells in transplantation therapy, many laboratory-to-clinic translational factors must first be considered, dynamics such as the source of the cells, ease of extraction, immunogenicity, capacity for proliferation, and cell yield [14]–[16]. These concerns may serve as potential limitations respective to the donor cell origin being considered, necessitating the need for a particular stem cell source to be more suitable for a specific disease.

Because stroke is a major cause of death and disability, any treatment that would help stroke patients recover some of the lost motor or cognitive function, would substantially improve their quality of life. Cell-based therapies have emerged as potential methods to treat several neuropathological diseases and injuries, including stroke [1]–[5], [9]–[13]. Laboratory studies and limited clinical trials have shown that transplantation of neural stem cells (NSCs) in stroke is safe and effective [17]–[20]. The mechanism of action of stem cell therapy for stroke remains not fully understood, but the two major postulated reparative pathways involve cell replacement and secretion of growth factors [1]–[5], [9]–[13], [21], [22]. To date, graft survival and integration with the host remain pressing concerns with cell-based treatment options. The current study set out to investigate those very issues using a human NSC line, NSI-566RSC, in a rat model of ischemic stroke.

Preclinical evidence has demonstrated the safety and efficacy of NSI-566RSC in animal models of the motor neuron disease amyotrophic lateral sclerosis (ALS) [23]–[26], spinal cord injury [27], and ischemic paraplegia [28]. Larger animal models have also been used to assess safety of NSI-566RSC for CNS transplantation [29], [30]. Functional recovery observed in these animal models has been ascribed to neuronal differentiation capacity of NSI-566RSC [25], [31], which parallels extensive *in vitro* characterization of these cells similarly demonstrating the cells’ ability to display neuronal phenotypic features (i.e., functional motoneurons) [32], [33]. The need for immunosuppression in order to augment graft survival and functional effects has been indicated in relevant ALS animal models [30], [34]. This translational research portfolio forms the basis for a clinical trial of transplanting NSI-566RSC in ALS patients [35].

Our long-standing interest in stem cell therapy for stroke prompted us to examine the efficacy of NSI-566RSC in an animal model of cerebral ischemia. We report here that intracerebral transplantation of NSI-566RSC at subacute phase of stroke produced behavioral improvements accompanied by graft survival and neuronal differentiation, extending the application of NSI-566RSC for transplantation therapy in ischemic stroke.

## Materials and Methods

### Subjects

All experiments were conducted in accordance with the National Institute of Health Guide and Use of Laboratory Animals, and were approved by the Institutional Animal Care and Use Committee of the University of South Florida, Morsani College of Medicine. Rats were housed two per cage in a temperature- and humidity-controlled room that was maintained on 12/12 hour-light/dark cycles. They had free access to food and water. All necessary steps were performed to minimize animal pain and stress throughout the study.

### Stroke and Transplant Surgery

A total of 32 male Sprague-Dawley rats received middle cerebral artery occlusion (MCAo), a well-established stroke model, as described previously [36]–[40]. Animals were anesthetized using isoflurane (1.5%–2.5% with oxygen). The scalp skin was shaved and scrubbed with alcohol and chlorhexidine surgical scrub. The animal was fixed in stereotaxic apparatus, then starting slightly behind the eyes, a midline sagittal incision about 2.5 cm long is made. With the rounded end of a spatula, the skull area was exposed. Using the bregma as reference point (i.e., prior to stroke surgery) laser Doppler recordings were obtained from the following coordinates (AP: +2.0, ML: ±2.0) at pre, during, and post surgery to reveal successful MCAo. The skin on the ventral neck was shaved from the jaw to the manubrium and scrubbed with alcohol and chlorhexidine surgical scrub. A skin incision was made over the right common carotid artery. The external carotid was isolated and ligated as far distally as possible. An incision using a pair of microscissor was made in the stump of the external carotid and a 4-0 nylon filament with a pre-fabricated end was inserted and passed up into the internal carotid artery until resistance was felt (approximately 15–17 mm), which effectively blocked the middle cerebral artery (MCA). The isoflurane was discontinued and the animal placed in a recovery cage over a warming blanket. After 60 minutes, the animal was anesthetized again with isoflurane and the incision opened. The filament causing the occlusion was removed and the stump of the external carotid ligated close to the carotid bifurcation. The skin incision was closed with staples. Finally, the animal was placed in a cage over a warming blanket until full recovery from anesthesia. Forty stroke animals were randomly divided into four Groups A–D (Table 1). They were tested for behavioral and neurological deficits on Day 6 post stroke (baseline), and then treated on Day 7 post stroke. Those animals that showed significant motor and neurological impairments were randomly assigned to receive NSI-566RSC intracerebral transplants at two sites within the striatum at three different doses: group A (0 cells/µl), group B (5,000 cells/µl), group C (10,000 cells/µl), and group D (20,000 cells/µl). The animal was fixed to a stereotaxic apparatus (Kopf Instruments). A 26-gauge Hamilton syringe was then lowered into a small burred skull opening (transplant coordinates were adjusted to 0.5 mm anterior and 2.8 mm lateral to bregma and 5.0 mm below the dural surface [41]. Within this single needle pass, 3 deposits of the test articles including 2 into the striatum (5.0 mm and 4.0 mm below the dural surface) and 1 into the cortex (3.0 mm below the dural surface) were made. The target area was the medial striatum and medial cortex which corresponds to the ischemic peri-infarct (or penumbra) area, based on previously established target sites for similar stereotaxic implants [42]. Each deposit consisted of 3 µl volume infused over a period of 3 minutes. All animals received daily immunosuppression (tacrolimus) 1 mg/kg/day IP. All animals were monitored weekly post-grafting for behavioral and neurological outcomes. Animals were euthanized at Day 56 days post-grafting and transcardially perfused with saline and 4% paraformaldehyde.

**Table 1:** Experimental Treatment Conditions.

Group	Group Size	Test Article	Dose	Survival Time
A	8	Vehicle	0 cells/µL×3 µl/site×3 sites	56+/−4 days
B	8	NSI-566RSC	5,000 cells/µL×3 µl/site×3 sites	56+/−4 days
C	8	NSI-566RSC	10,000 cells/µL×3 µl/site×3 sites	56+/−4 days
D	8	NSI-566RSC	20,000 cells/µL×3 µl/site×3 sites	56+/−4 days

Treatment Summary Table. Table 1 summarizes the 4 treatment groups of randomly assigned stroke animals.

### Behavioral Tests

All investigators testing the animals were blinded to the treatment condition. Animals were subjected to elevated body swing test (EBST) and neurological exam. EBST involved handling the animal by its tail and recording the direction of the swings [36]–[40]. The test apparatus consisted of a clear Plexiglas box (40×40×35.5 cm). The animal was gently picked up at the base of the tail, and elevated by the tail until the animal’s nose was at a height of 2 inches (5 cm) above the surface. The direction of the swing, either left or right, was counted once the animals head moved sideways approximately 10 degrees from the midline position of the body. After a single swing, the animal was placed back in the Plexiglas box and allowed to move freely for 30 seconds prior to retesting. These steps were repeated 20 times for each animal. Intact rats displayed a 50% swing bias, that was, the same number of swings to the left and to the right. A 75% swing bias was used as criterion of successful MCAo. About one hour after the EBST, neurological exam was conducted as previously described [36]–[40] with minor modifications. Neurologic score for each rat was obtained using 3 tests which included (1) forelimb retraction, which measures the ability of the animal to replace the forelimb after it was displaced laterally by 2 to 3 cm, graded from 0 (immediate replacement) to 3 (replacement after several seconds or no replacement); (2) beam walking ability, graded 0 for a rat that readily traversed a 2.4-cm-wide, 80-cm-long beam to 3 for a rat unable to stay on the beam for 10 seconds; and (3) bilateral forepaw grasp, which measured the ability to hold onto a 2-mm-diameter steel rod, graded 0 for a rat with normal forepaw grasping behavior to 3 for a rat unable to grasp with the forepaws. The scores from all 3 tests were averaged to give a mean neurologic deficit score (maximum possible score, 9 points divided by 3 tests = 3). Animals were subjected to both tests at baseline (prior to stroke), then at 7 days after stroke (prior to transplantation) and at weekly intervals over an eight-week period.

### Histology

All histological analyses were performed at Neuralstem Inc. (San Diego, CA). Brains were cryoprotected in PBS containing 30% sucrose and later sectioned at 40 µm thickness using a freezing sliding microtome. Every sixth section through the brain was selected for staining.

### Immunohistochemistry/Histology

Sections were treated with 3% hydrogen peroxide for 15 minutes, washed 3 times with PBS, and further treated with 0.3% Triton X-100 for 30 minutes followed by wash. Tissue was then blocked in normal horse serum for 90 minutes, and incubated with either mouse anti human nuclei (HuNu; MAB1281; Chemicon International, Temecula, CA; 1∶2000) or chicken anti human-specific neuron-specific enolase (hNSE; AB-9698; Chemicon International, Temecula, CA 1∶2000) at room temperature overnight. Sections were washed and again blocked with horse serum for 90 minutes, then treated with biotinylated donkey anti-mouse or biotinylated donkey anti-chicken secondary antibody (Jackson ImmunoResearch, West Grove, PA; 1∶2000) for 90 minutes. Following wash, sections were incubated with streptavidin conjugated to horseradish peroxidase (Jackson ImmunoResearch, West Grove, PA; 1∶5000) for 90 minutes, and then developed with diaminobenzidine containing nickel chloride for 1 minute. The sections were then mounted on gelatin-coated slides, dried, labeled, and cover-slipped. In addition, sections adjacent to sections (1-in-6 series) stained for human stem cells were processed with hematoxylin and eosin (H&E). All slides were examined and scanned using Nikon AZ100 Multizoom microscope (Nikon Instruments, Inc, Japan) for further qualitative and semi-quantitative analyses.

### Measurements of Infarct Size

At least 4 coronal sections per brain were processed for H&E. The indirect lesion area, in which the intact area of the ipsilateral hemisphere was subtracted from the area of the contralateral hemisphere, was calculated to reveal cerebral infarction. Scores for infarct lesion were rated accordingly: LOW <30%, MEDIUM 30–60%, HIGH >60%, while scores for volume were rated as follows: Low <1 mm^3^, Medium 1–2 mm^3^, High >2 mm^3^. For calculation of area of infarction in mm2, a separate cohort of animals (n = 8 per group) subjected to the same experimental paradigm were processed for Triphenyltetrazolium chloride (TTC) staining. Anesthetized animals were perfused intracardially with saline. The brain tissue was then removed, immersed in cold saline for 5 minutes, and sliced into 2.0-mm thick sections. The brain slices were incubated in a 2% triphenyltetrazolium chloride (Sigma) dissolved in normal saline for 30 minutes at 37°C and then transferred into a 5% formaldehyde solution for fixation. The area of infarction in each slice was measured using a digital scanner and the Image Tools program (University of Texas Health Sciences Center in San Antonio). The area of infarction in each animal was obtained from largest infarcted brain slice.

### Cell Engraftment and Neuronal Phenotype Assessment

The slides were examined at 10X magnification of Nikon AZ100 Multizoom microscope and digitized using a PC-based Image Tools computer program (Nikon, NIS Elements software). Brain sections were blind-coded in assessing cell engraftment index for NSI-566RSC graft survival based on HuNu stained and hNSE stained slides, respectively.

### Statistical Analysis

The data were evaluated statistically using either Two-way analysis of variance (ANOVA), or repeated measure of ANOVA and subsequent post hoc compromised t-tests for behavior. These statistical analyses employed a StatView 5.0 software (USA). Statistical significance was preset at p<0.05.

## Results

### NSI-566RSC Cell Grafts Promote Behavioral Recovery in Stroke Animals

**Figure 1:**
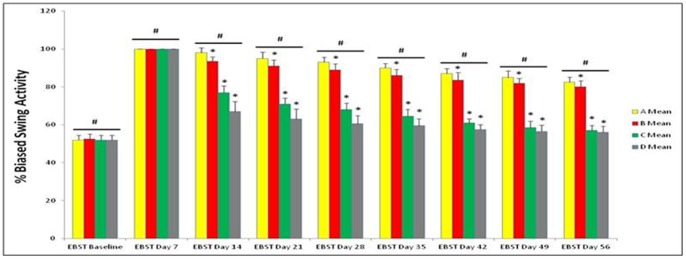
NSI-566RSC cell grafts ameliorate stroke-induced motor deficits. Motor performance was measured by EBST (Figure 1). All animals displayed normal motor behavior at Baseline (i.e., prior to stroke). At day 7 post-stroke, all animals exhibited 100% biased swing behaviors, indicating that all animals received successful stroke. At 14 days post-stroke onwards, dose-dependent and timing-dependent effects of the treatment were recognized, in that the improvement in behavioral performance was in the order of high dose to zero dose as follows: 20,000 cells/µl (D) >10,000 cells/µl (C) >5,000 cells/µl (B)>vehicle infusion only (A). In addition, over time there was a trend of better improvement, with the most significant improvement seen at 56 days post-stroke. *significant <0.05 vs. other treatment groups within time point; ^#^significant <0.05 vs. other time points.

**Figure 2:**
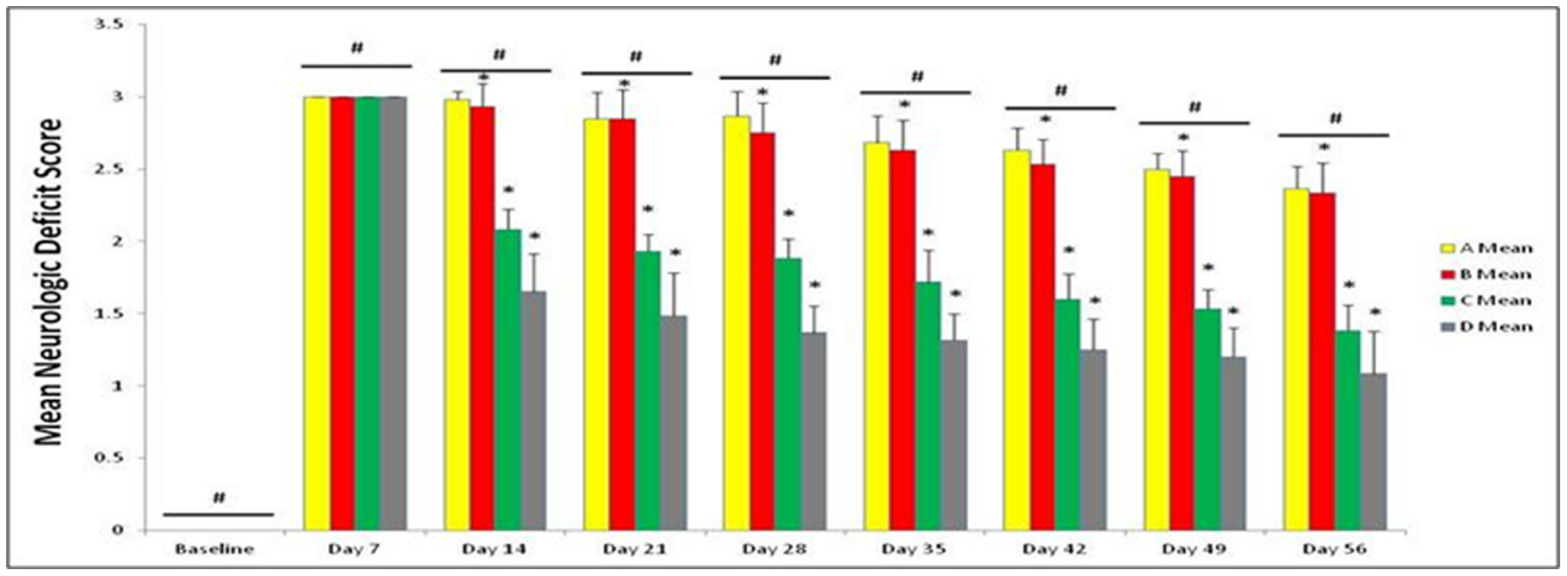
NSI-566RSC cell grafts attenuate stroke-induced neurologic impairments. Neurological function was assessed using a battery of neurological tests (Figure 2). All animals displayed normal neurological function at Baseline (i.e., prior to stroke). At day 7 post-stroke, all animals exhibited significant impairment in neurological function, indicating that all animals received successful stroke. At 14 days post-stroke onwards, dose-dependent and timing-dependent effects of the treatment were recognized, in that the improvement in neurological performance was in the order of high dose to zero dose as follows: 20,000 cells/µl (D) >10,000 cells/µl (C) >5,000 cells/µl (B)>vehicle infusion only (A). In addition, over time there was a trend of better improvement, with the most significant improvement seen at 56 days post-stroke. *significant <0.05 vs. other treatment groups within time point; **^#^**significant <0.05 vs. other time points.

Behavioral analyses revealed that all animals included in this study displayed normal behaviors at baseline prior to stroke surgery. At 7 days post-stroke, all animals exhibited the typical stroke-induced behavioral deficits characterized by significantly biased swing activity and impaired neurological performance. However, at 14 days post-stroke (i.e., 7 days post-transplantation onwards), significant recovery of motor (Figure 1) and neurological (Figure 2) functions in stroke animals that received intracerebral transplantation of high doses of 10,000 cells/µl (C) and 20,000 cells/µl (D) of NSI-566RSC compared to vehicle-infused stroke animals (A) or those stroke animals that received the low dose of 5,000 cells/µl (B). ANOVA revealed statistically significant treatment effects (p’s<0.01). Post hoc compromised t-tests also revealed dose-dependent treatment effects and post-transplantation timing effects (p’s<0.05). There was stable improvement in those stroke transplanted with high doses of cells (Groups C and D) which showed a trend of better improvement over time up to the 56-day study period. Although there was slight improvement in the behavioral performance of vehicle-infused stroke animals and stroke animals that received the low dose of 5,000 cells/µl, the recovery of motor and neurological tests demonstrated by high dose transplanted stroke animals was significantly better throughout the 56-day period compared to vehicle-infused stroke animals or those stroke animals that received the low dose of 5,000 cells/µl (p’s<0.05). There were significant dose-dependent differences in the behavioral improvement across treatment groups at post-transplantation periods with the highest dose of 20,000 cells/µl showing the most significant improvement in both motor and neurological tests (p’s<0.05). Similarly, there were significant differences in the behavioral performance among treatment groups at post-transplantation periods with the most significant improvement in both motor and neurological tests seen at day 56 post-transplantation (p’s<0.05).

### NSI-566RSC Cell Grafts do not Reduce Infarct Size

**Figure 3:**
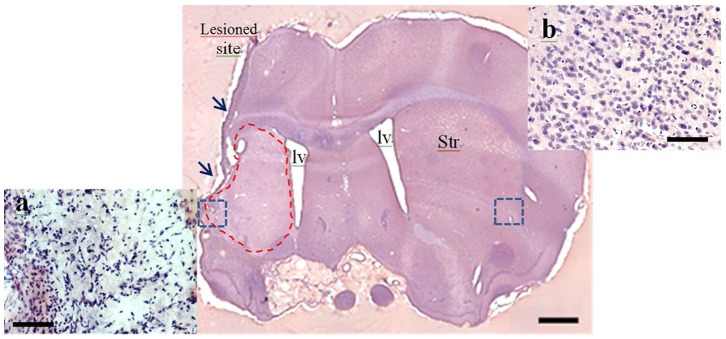
A typical infarct lesion in the striatum and cortex. The tissue damage covers the cortex and infarct striatum (dash area) and areas lateral to infarct core lesion (not shown here). Note the shrinkage and dissolution of the cells in the penumbra (box) compared to the corresponding contralateral side of the brain (Insets a and b). Scale bar = 1.0 mm [Iv; lateral ventricles, Str: striatum].

To confirm infarcts in stroke animals, H&E was used. Infarcts were confirmed in 93% of the animals; infarct size was similar across all groups (*p* = 0.2676) (Figure 3). A summary of infarct frequency for each treatment group is presented for clarity (Table 2). Additionally, we rated the size of the infarct in all animals to get a better approximation of any infarct variability that might have been affected by the grafts, but did not detect any as significant number of outliers across the treatment groups (i.e., only 1 animal each from Groups A–C). Based on these data, infarct size was not reduced by NSI-566RSC cell grafts.

**Table 2:** Summary of histological assessment of infarct brains transplanted with NSI-566RSC (n = 10 per group)*.

	Stains	Group A-Vehicle	Group B-Low	Group C-Medium	Group D-High	Mean-Total
Freq. of the infarct lesion**	*H&E*	9 (90%)	9 (90%)	9 (90%)	10 (100%)	93%
Freq. of surviving grafts in the striatum	*HuNu*	Not present	10 (100%)	9 (90%)	9 (90%)	93%
	*hNSE*	Not present	10 (100%)	9 (90%)	8 (80%)	90%
Est. graft size (mm^3^)	*HuNu*	Not present	0.85±0.37	0.19±0.05	0.63±0.22	0.56±0.15
	*hNSE*	Not present	0.99±0.50	0.16±0.04	0.54±0.33	0.58±0.21

Table 2 summarizes the histological assessment of infarct brains that received three different doses of NSI-566RSC cell lines (Group B, C, and D) in comparison with the vehicle (Group A).

*Quantitative analysis did not find statistical difference in graft size across the three dose groups, either by HuNu or by hNSE stain (*p* = 0.2002).

**Freq. of infarct lesion was based on evidence of tissue damage and cellular abnormality such as cell loss, apoptotic bodies in penumbra regions.

### NSI-566RSC Cell Grafts Survive and Display Neuronal Phenotype

**Figure 4:**
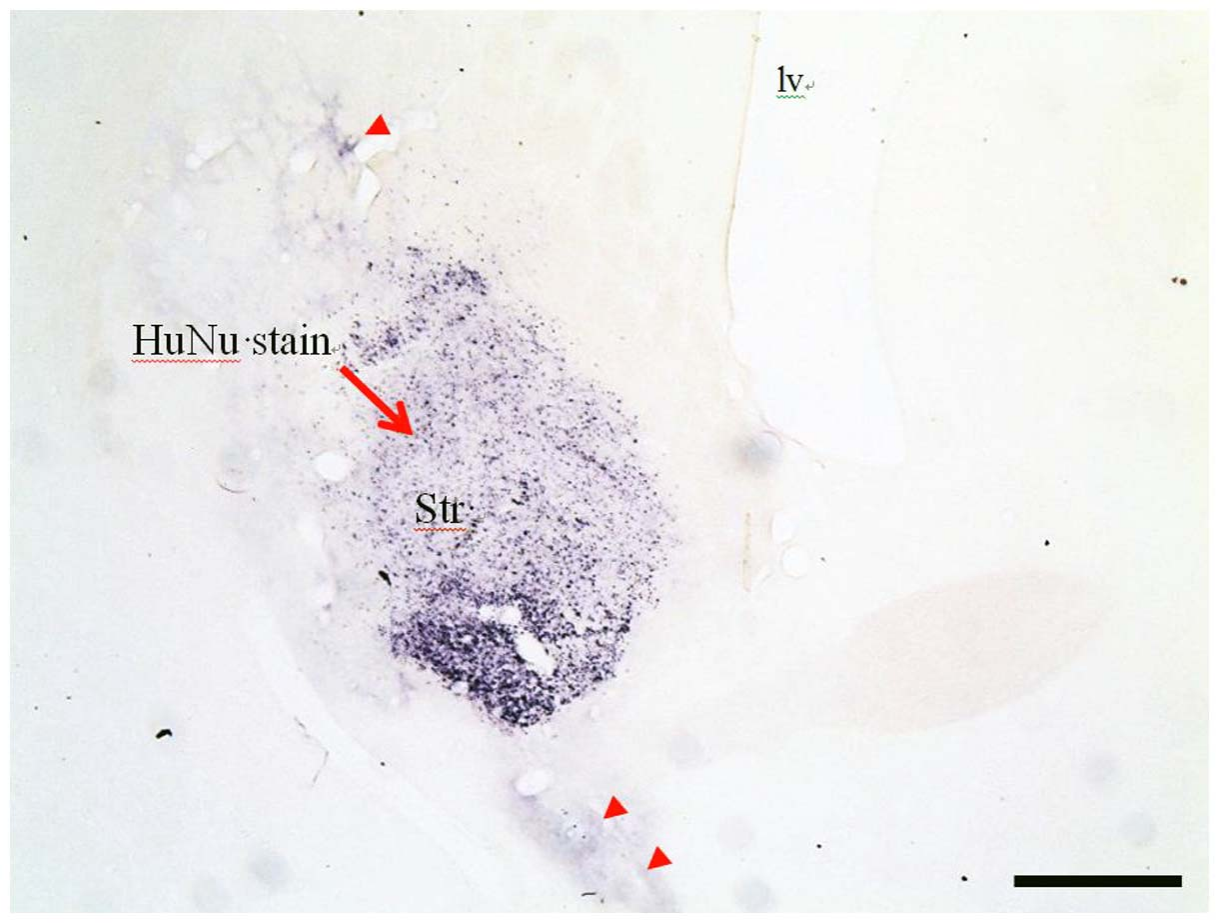
A representative image of HuNu staining in the striatum. HuNu+cells were confined to the striatum of rats receiving the lowest dose of graft cells (Group B). Note that small amounts of HuNu staining extended both dorsally and ventrally from the striatum. Scale bar = 0.5 mm [Iv; lateral ventricle, Str: striatum].

**Figure 5:**
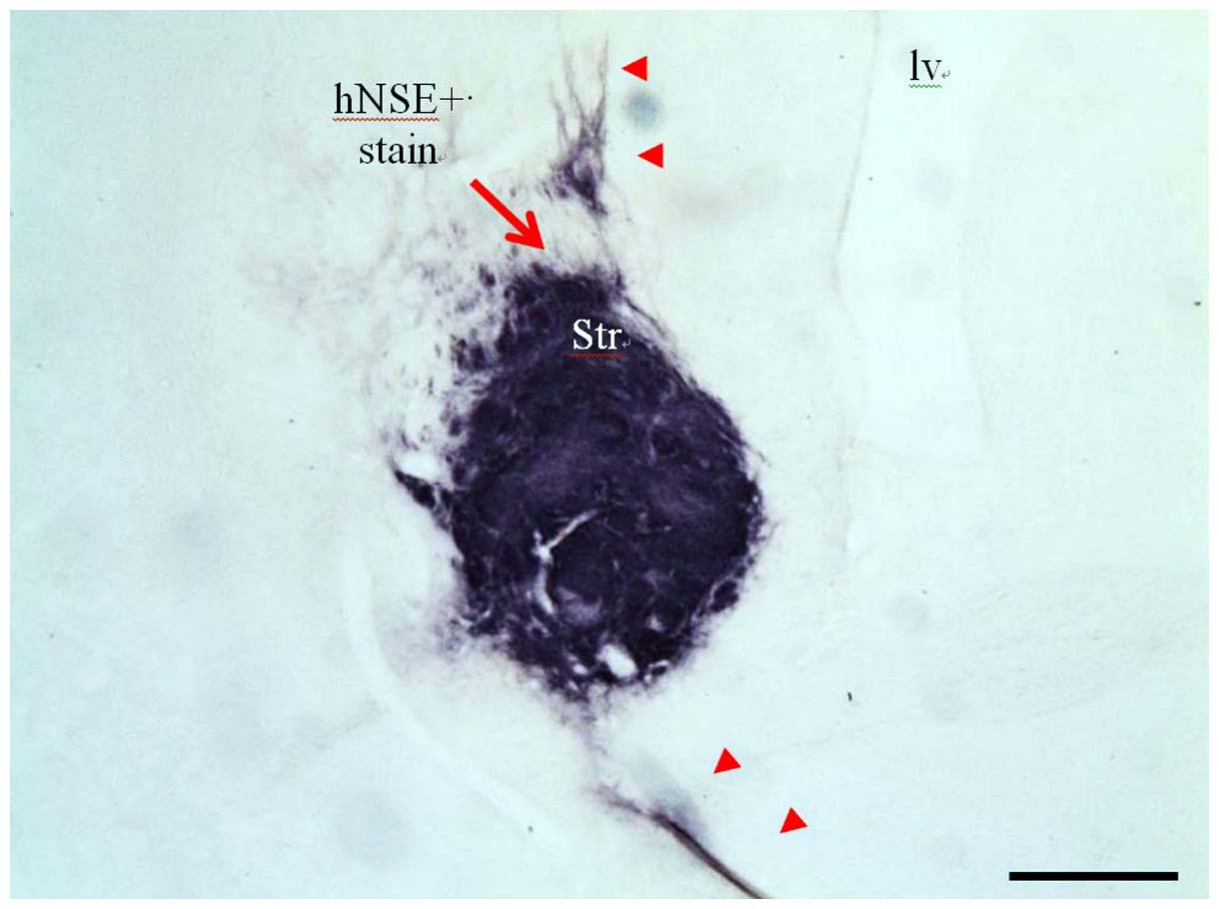
A representative image of hNSE staining in the striatum. Human graft cells were densely confined to the striatum (arrow) with small amounts of hNSE+fibers that extended dorsally and ventrally from the striatum (arrowheads). Scale bar = 0.5 mm [Iv; lateral ventricle, Str: striatum].

**Figure 6:**
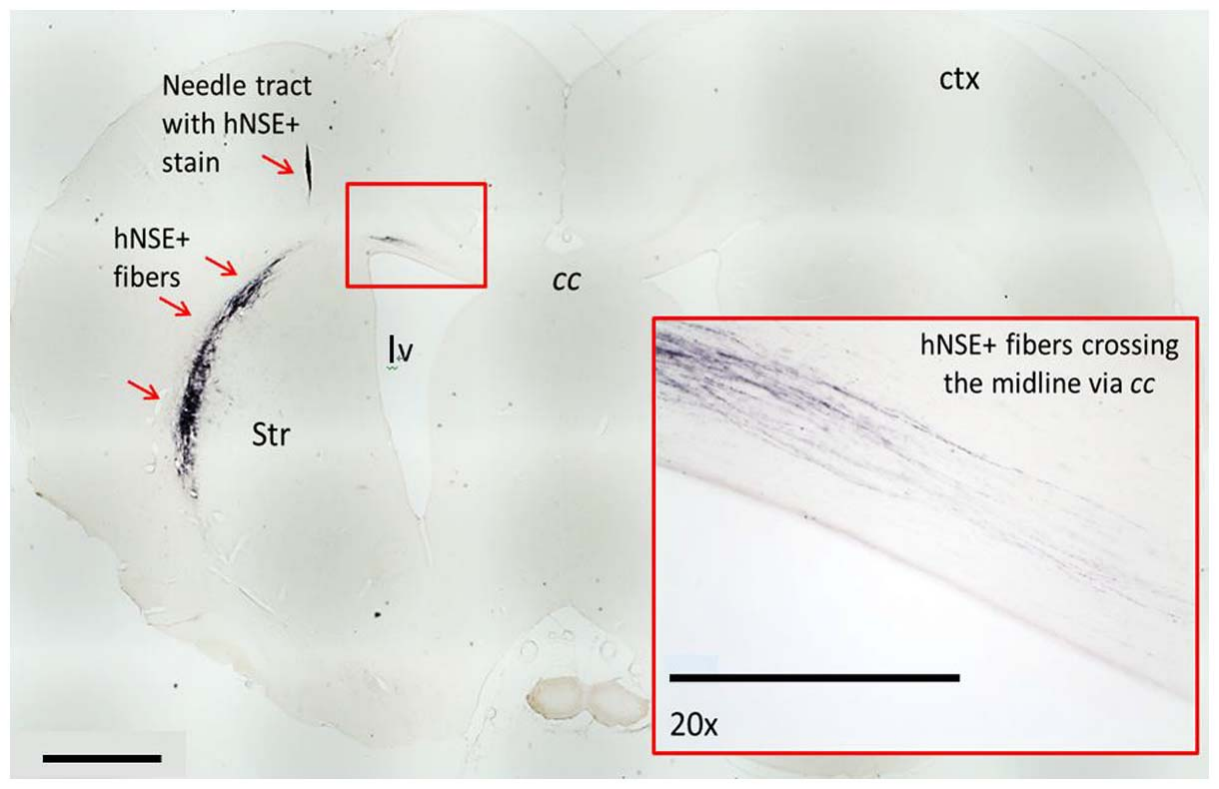
Migration of hNSE+fibers toward the contralateral side of non-infarct region. Needle tract with hNSE+stain was found in the cortex. Additionally, small amounts of hNSE+fibers crossed the *cc contralaterally (inset).* Scale bar = 1.0 mm [Iv; lateral ventricle, Str: striatum, ctx: cortex, *cc*: corpus callosum].

To identify the cell graft, and estimate volume, immunohistochemistry was performed using two human-specific antibodies: one to detect all HuNu (Figures 4 & 5), and another to detect hNSE (Figure 6). Tabular summaries of the frequency of surviving grafts and estimated graft size (Table 2 and Table 3) are presented for clarity. Surviving cell grafts were confirmed in 10/10 animals of group A, 9/10 group B, and 9/10 in group C. hNSE and HuNu staining revealed similar graft volume estimates (HuNu volume: 0.56±0.15 *mm* ^3^ and hNSE volume: 0.58±0.21 *mm* ^3^). hNSE-immunoreactive fibers were also present within the corpus callosum, coursing in parallel with host tracts, suggesting a propensity to follow established neuroanatomical features. Supplementary files are also provided to show individual animal’s histology capturing representative images of HuNu and hNSE (Figures S1, S2, S3, S4, S5, S6, S7, S8, and S9).

**Table 3:** Summary of infarct size and graft size.

ID	H&E-infarct	HuNu-Volume	hNSE-Volume
**Group A Vehicle**	STR+CTX	STR+CTX	STR+CTX
A02	LOW	None	None
A04	LOW	None	None
A13	LOW	None	None
A14	LOW	None	None
A15	HIGH	None	None
A18	LOW	None	None
A19	LOW	None	None
A20	LOW	None	None
A25	LOW	None	None
A26	None	None	None
**Group B (5** **K cells/µl)**			
B01	None	LOW	LOW
B03	LOW	LOW	LOW
B05	HIGH	HIGH	HIGH
B06	MEDIUM	HIGH	LOW
B07	LOW	LOW	LOW
B08	LOW	LOW	LOW
B09	LOW	LOW	LOW
B10	LOW	HIGH	HIGH
B11	LOW	LOW	LOW
B12	LOW	LOW	MEDIUM
**Group C (10** **K cells/µl)**			
C21	LOW	LOW	LOW
C22	LOW	LOW	LOW
C23	LOW	LOW	LOW
C24	MEDIUM	LOW	LOW
C01	LOW	LOW	LOW
C02	LOW	LOW	LOW
C06	MEDIUM	LOW	LOW
C27	LOW	LOW	LOW
C28	LOW	LOW	N/A
C29	None	LOW	LOW
**Group D** **(20 K cells/µl)**			
D05	LOW	LOW	LOW
D08	LOW	None	None
D11	HIGH	MEDIUM	HIGH
D16	HIGH	HIGH	LOW
D19	MEDIUM	LOW	LOW
D21	LOW	LOW	LOW
D22	MEDIUM	LOW	LOW
D23	LOW	LOW	LOW
D24	MEDIUM	LOW	LOW
D30	LOW	None	None

Table 3 summarizes the semi-quantitative rating of the infarct size and the size of HuNu (nuclei staining) or hNSE (neuropil staining) positive grafts.

1. Scores for Infarct lesion: LOW <30%, MEDIUM 30–60%, HIGH >60%.

2. Scores for Volume: Low <1 mm^3^, Medium 1–2 mm^3^, High >2 mm^3^.

3. Abbreviations: STR: striatum, CTX: cerebral cortex.

## Discussion

Here, we demonstrated that intracerebral transplantation of NSI-566RSC, a spinal cord-derived NSC line, reduced behavioral deficits associated with ischemic stroke. Significant improvements in both motor and neurological tests were detected in the NSI-566RSC-treated stroke animals. In addition, the results revealed significant dose-dependent differences in the behavioral improvement across treatment groups at post-transplantation periods with the highest NSI-566RSC dose showing the most significant improvement in both motor and neurological tests. Similarly, there were significant differences in the timing of behavioral performance among treatment groups at post-transplantation periods with the most significant improvement in both motor and neurological tests seen at day 56 post-transplantation, indicating that the transplanted stroke animals showed improved recovery over graft maturation period. We observed though that a few transplanted animals did not contain surviving grafts. The reason why some transplanted animals do not have detectable transplanted cells is unknown but it is likely because of graft failure due to immunosuppressant being ineffective to those particular animals.

We used a potent immunosuppressive agent (tacrolimus) to avoid rejection of the human cells, but are aware of the action of this drug in the brain response to ischemia itself or a possible synergic effect with the cell-based therapy proposed in our study. However, in our previous studies, using cyclosporine and tacrolimus [43]–[45], we came into the conclusion that these immunosuppressant drugs, albeit with neuoroprotective effects, display limited therapeutic window of opportunity, in that the drugs must be administered prior to, during, and/or immediately after the injury. Beyond this very acute time point, such as the present two weeks post-stroke period, we and several others have found that these drugs are not effective. In addition, a vis-à-vis comparison between non-immunosuppressed transplanted stroke animals versus immunosuppressed transplanted stroke animals revealed that immunosuppressed transplanted stroke animals significantly produced functional recovery compared to immunosuppression treatment alone [46]. Moreover, the choice to deliver the cocktail of immunosuppressants in the present study was based on a series of preclinical studies that supported the ongoing clinical trials of the same cells in ALS patients. This cocktail was proven effective in facilitating the graft effects in animal models of ALS, and approved by the FDA for this clinical trial. Accordingly, as we translate these NSCs for stroke clinical application, in keeping with the FDA’s requirement and also based on preclinical studies from our group and several others, we maintain the transplant protocol with the immunosuppresion regimen. However, if the transplant procedure was done acutely in stroke, we would be compelled to conduct another vis-à-vis study comparing NSCs with vehicle versus NSCs with immunosuppressants. For the present delayed transplantation regimen, and with the FDA requiring that the same protocol done in ALS be followed in the stroke arena in order to gain entry into the clinic, both scientific and clinical bases support our decision to maintain the NSCs with immunosuppressant regimen.

This study was designed to evaluate the potential therapeutic value of intracerebral dosing of NSI-566RSC in an animal model of adult ischemic stroke. The recovery of motor and neurological tests demonstrated by high dose transplanted stroke animals was significantly better throughout the 56 day study period compared to vehicle-infused stroke animals, or low-dosed transplanted animals. Moreover, there was stable improvement in the high dose animals, and they showed a trend of better improvement over time.

These results demonstrated safety and efficacy of NSI-566RSC in a subacute model of ischemic stroke in rats, indicating a strong proof-of-principle data that NSI-566RSC are potent cell donors for transplantation therapy to treat paralysis in stroke patients. A Phase I trial testing the safety of NSI-566RSC has recently been completed in the treatment of ALS [35]. Our current observations of safety and efficacy of NSI-566RSC in stroke animals mimic similar therapeutic readouts following transplantation of NSI-566RSC in animal models of ALS [23]–[26], spinal cord injury [27], and ischemic paraplegia [28]. Testing the efficacy of NSI-566RSC in larger stroke animals may be needed, although a safety portfolio already exists in mini-pigs for NSI-566RSC transplantation in CNS [29], [30]. That the present transplanted stroke animals exhibited improvement in motor and neurological functions accompanied by NSI-566RSC graft survival and neuronal differentiation also resembled the histopathological outcomes seen in animal models of ALS, spinal cord injury, ischemic paraplegia demonstrating engraftment and neuronal phenotypic persistence in transplanted NSI-566RSC [25], [31]. While graft survival and neuronal differentiation have been confirmed, on-going work is investigating the integration of graft into host tissue, determining the neuronal phenotypes of the grafted neurons, and confirming the low proliferation rates of the grafts as suggested from these preliminary histological studies.

Stroke remains a significant unmet clinical need [1]–[5] with tPA efficacy limited to 4.5 hours after stroke onset and benefits only about 3% of ischemic stroke patients [6]–[8]. The tPA’s narrow therapetuc window has prompted the need for novel treatments, such as stem cell therapy, designed to abrogate stroke beyond the acute phase of the disease [9]–[13]. Current clinical trials of cell therapy for stroke have pursued adult stem cells (.e.g., bone marrow and placenta) and NSCs, based on ample laboratory evidence demonstrating safety and efficacy of these cells [4], [5], [42], [47]–[49]. Optimization of clinical outcomes has been a key translational research effort for cell therapy for stroke [10], [13], [50]–[52]. With myriad stem cell sources in transplantation therapy, many laboratory-to-clinic translational factors have been recognized including well-defined donor stem cells, cell isolation and propagation, immunogenicity, capacity for proliferation, and cell yield [14]–[16]. Although NSC transplantation has been shown effective in stroke models [19], [53], [54], the mechanism of action remains elusive, in particular graft survival and integration with the host have yet to be fully understood in the stroke brain. Here, we provide evidence of a regenerative pathway involving robust NSI-566RSC engraftment and neuronal differentiation that accompanied behavioral improvements despite absence of cerebral infarct reduction. Axonal growth with long fibers from NSC grafts extended to the corpus callosum and traversed with host tracts, indicating graft-host integration recapitulating endogenous neuroanatomical landmarks. In addition, while on-going clinical trials of cell therapy have targeted the acute and chronic phase of stroke, the present study suggests the sub-acute phase as an equally effective therapeutic window for NSI-566RSC transplantation in ischemic stroke.

## Supporting Information

Figure S1 **HuNu staining in vehicle-infused group.** No detectable HuNu staining is observed.
(TIF)

Figure S2 **HuNu staining in low dose-transplanted group.** HuNu staining is detected in all transplanted stroke brain.
(TIF)

Figure S3 **HuNu staining in medium dose-transplanted group.** HuNu staining is detected in all transplanted stroke brain except for one brain (C29).
(TIF)

Figure S4 **HuNu staining in high dose-transplanted group.** HuNu staining is detected in all transplanted stroke brain except for one brain (D30).
(TIF)

Figure S5 **hNSE staining in vehicle-infused group.** No detectable hNSE staining is observed.
(TIF)

Figure S6 **hNSE staining in low dose-transplanted group.** hNSE staining is detected in all transplanted stroke brain.
(TIF)

Figure S7 **hNSE staining in medium dose-transplanted group.** hNSE staining is detected in all transplanted stroke brain except for one brain (C29).
(TIF)

Figure S8 **hNSE staining in high dose-transplanted group.** hNSE staining is detected in all transplanted stroke brain except for two brains (D08 and D30).
(TIF)

Figure S9 **Area of infarction.** TTC staining reveals area of infarction (mm^2^) in the laragest infarcted slice. *p<0.05 versus A or B.
(TIF)
