# Supplementary figures and images for: Behavioral and Histopathological Assessment of Adult Ischemic Rat Brains after Intracerebral Transplantation of NSI-566RSC Cell Lines
Source: PLoS One. 2014 Mar 10;9(3):e91408. doi: 10.1371/journal.pone.0091408 (PMC3948841; doi:10.1371/journal.pone.0091408)

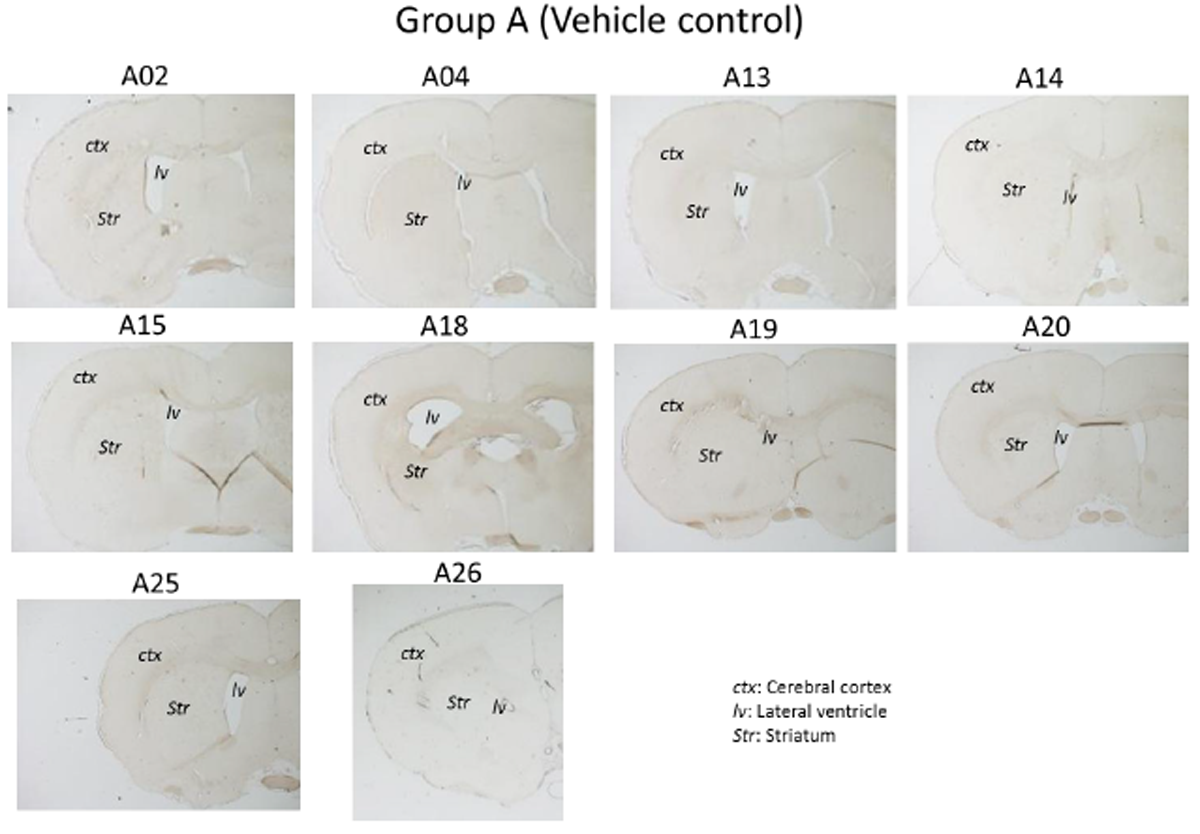

Supplement: Figure S1 — HuNu staining in vehicle-infused group. No detectable HuNu staining is observed. (TIF) [file pone.0091408.s001.tif]

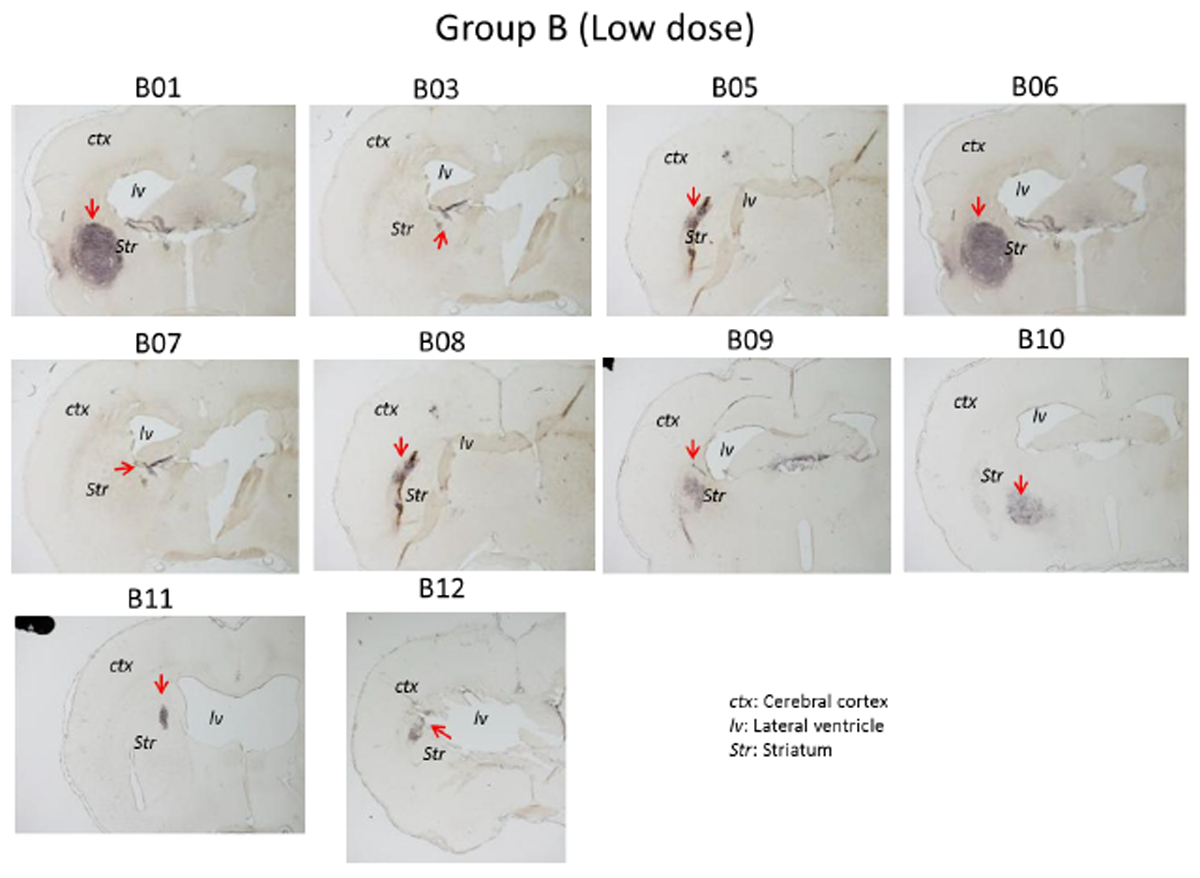

Supplement: Figure S2 — HuNu staining in low dose-transplanted group. HuNu staining is detected in all transplanted stroke brain. (TIF) [file pone.0091408.s002.tif]

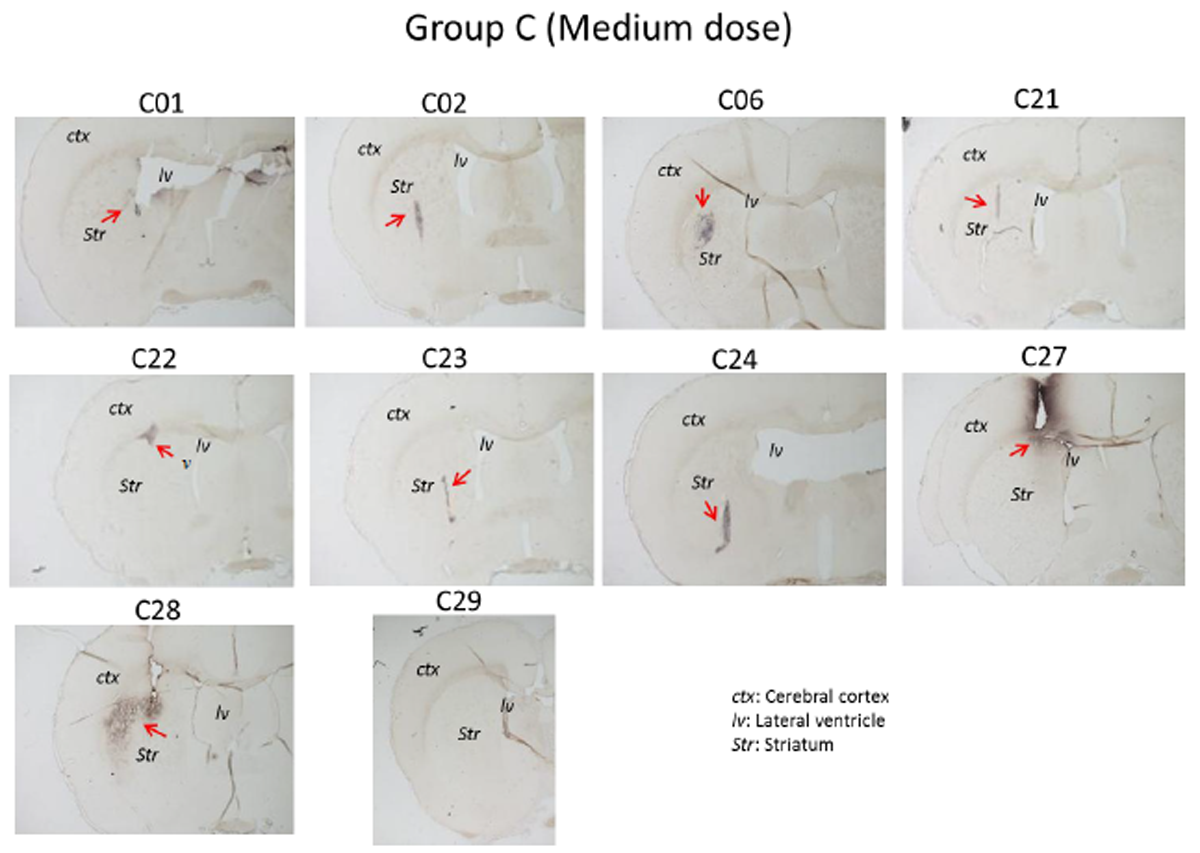

Supplement: Figure S3 — HuNu staining in medium dose-transplanted group. HuNu staining is detected in all transplanted stroke brain except for one brain (C29). (TIF) [file pone.0091408.s003.tif]

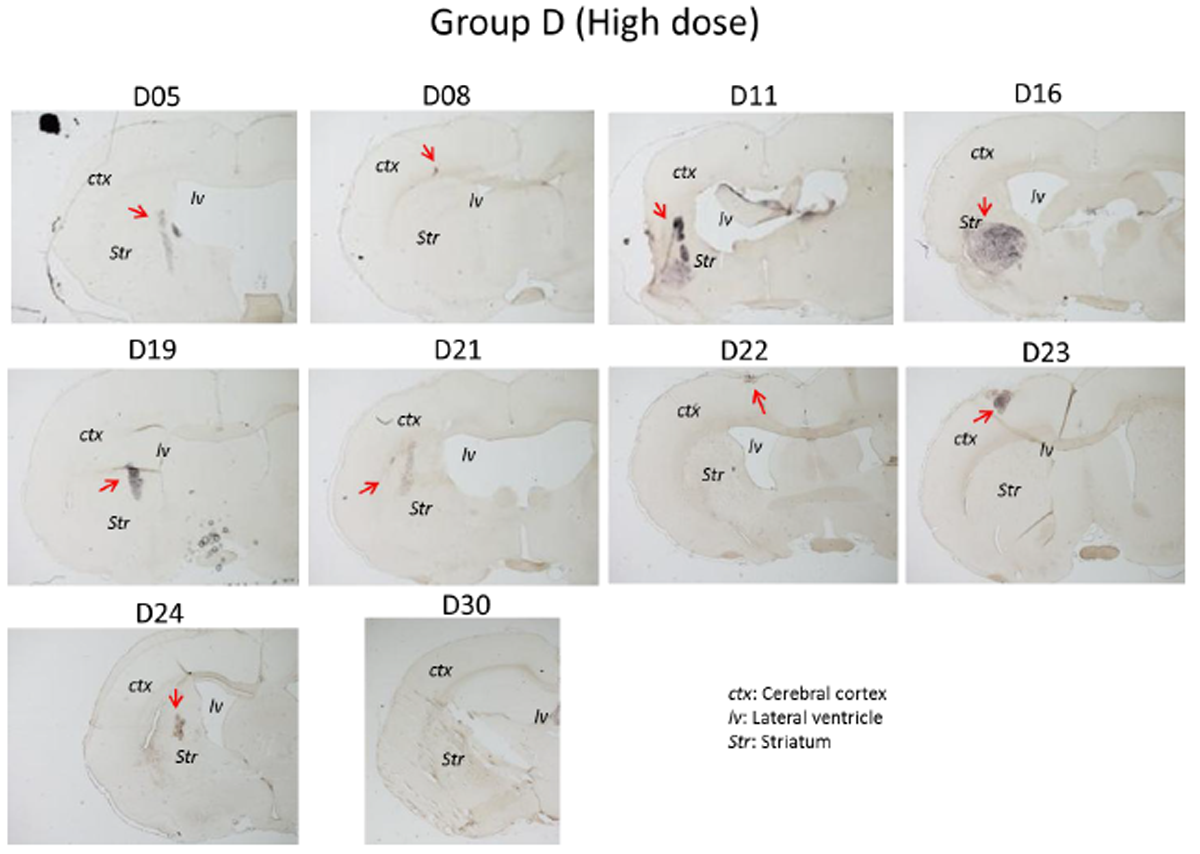

Supplement: Figure S4 — HuNu staining in high dose-transplanted group. HuNu staining is detected in all transplanted stroke brain except for one brain (D30). (TIF) [file pone.0091408.s004.tif]

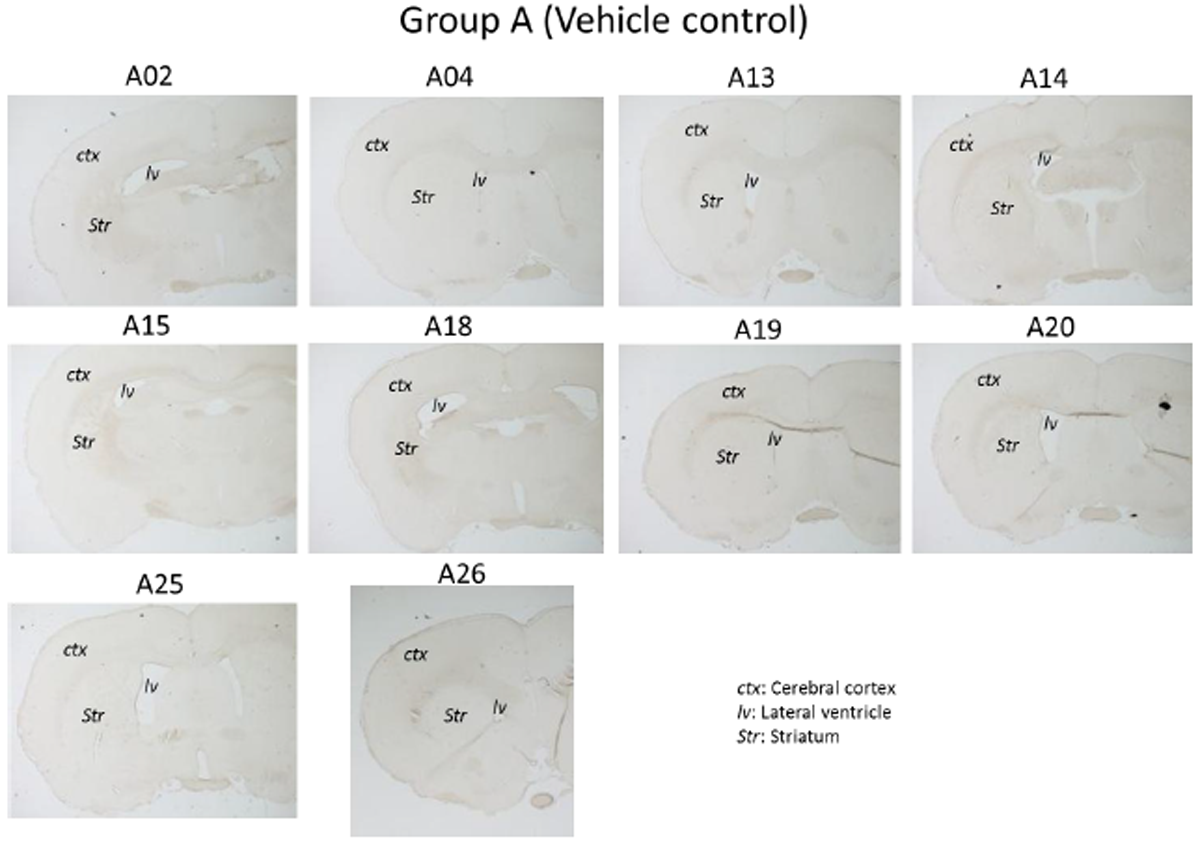

Supplement: Figure S5 — hNSE staining in vehicle-infused group. No detectable hNSE staining is observed. (TIF) [file pone.0091408.s005.tif]

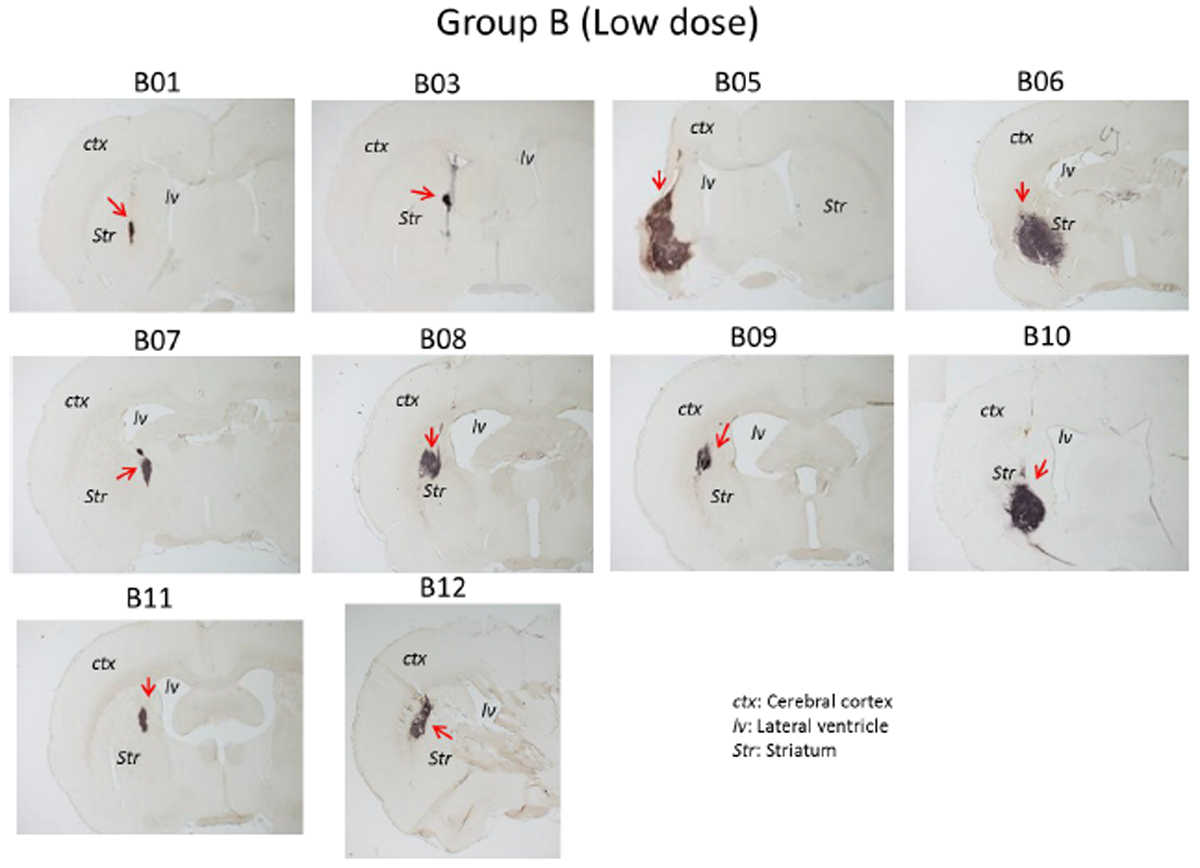

Supplement: Figure S6 — hNSE staining in low dose-transplanted group. hNSE staining is detected in all transplanted stroke brain. (TIF) [file pone.0091408.s006.tif]

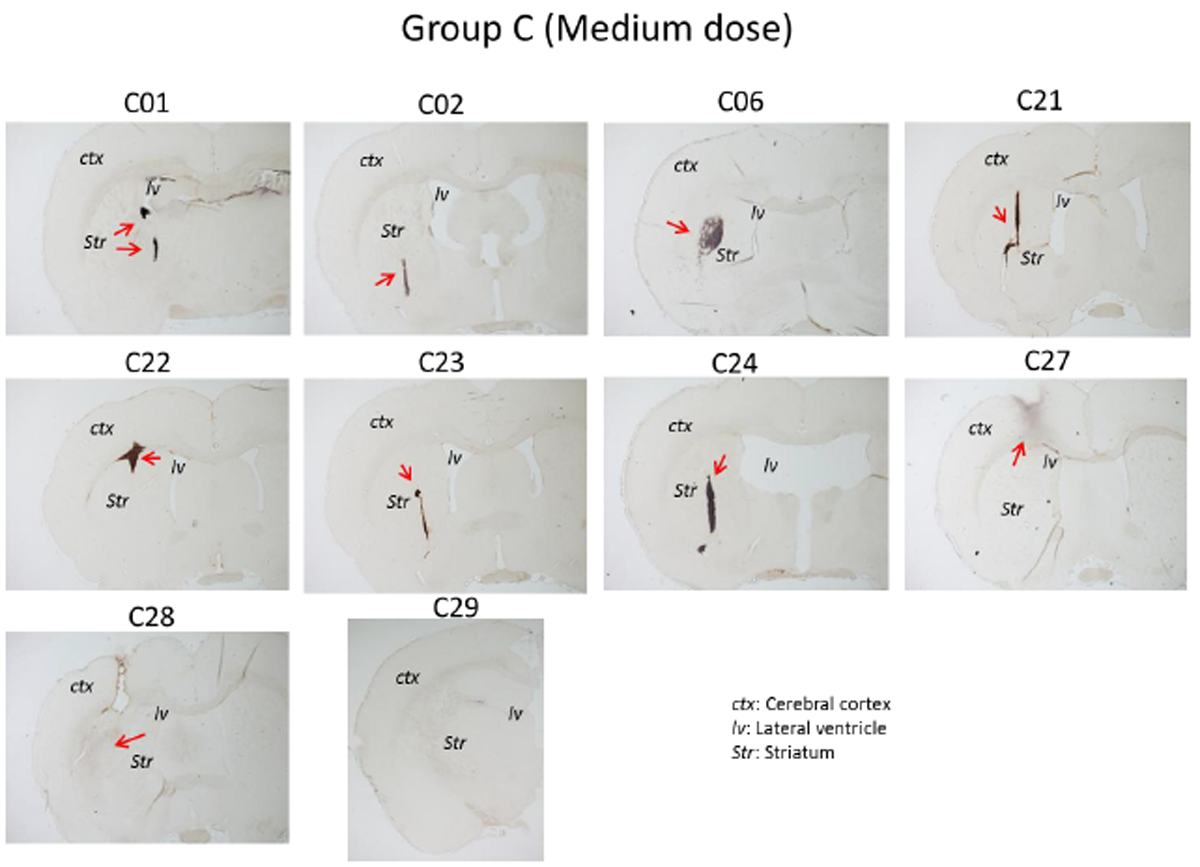

Supplement: Figure S7 — hNSE staining in medium dose-transplanted group. hNSE staining is detected in all transplanted stroke brain except for one brain (C29). (TIF) [file pone.0091408.s007.tif]

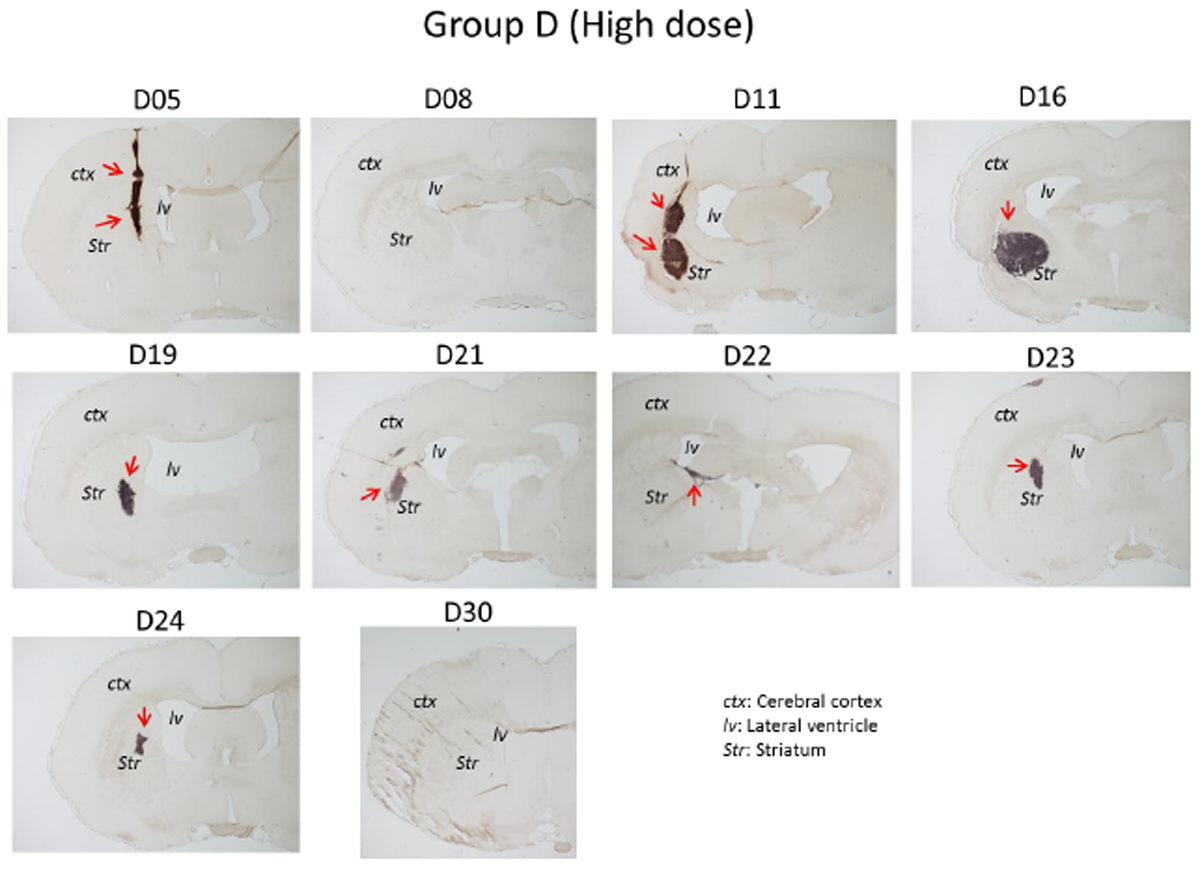

Supplement: Figure S8 — hNSE staining in high dose-transplanted group. hNSE staining is detected in all transplanted stroke brain except for two brains (D08 and D30). (TIF) [file pone.0091408.s008.tif]

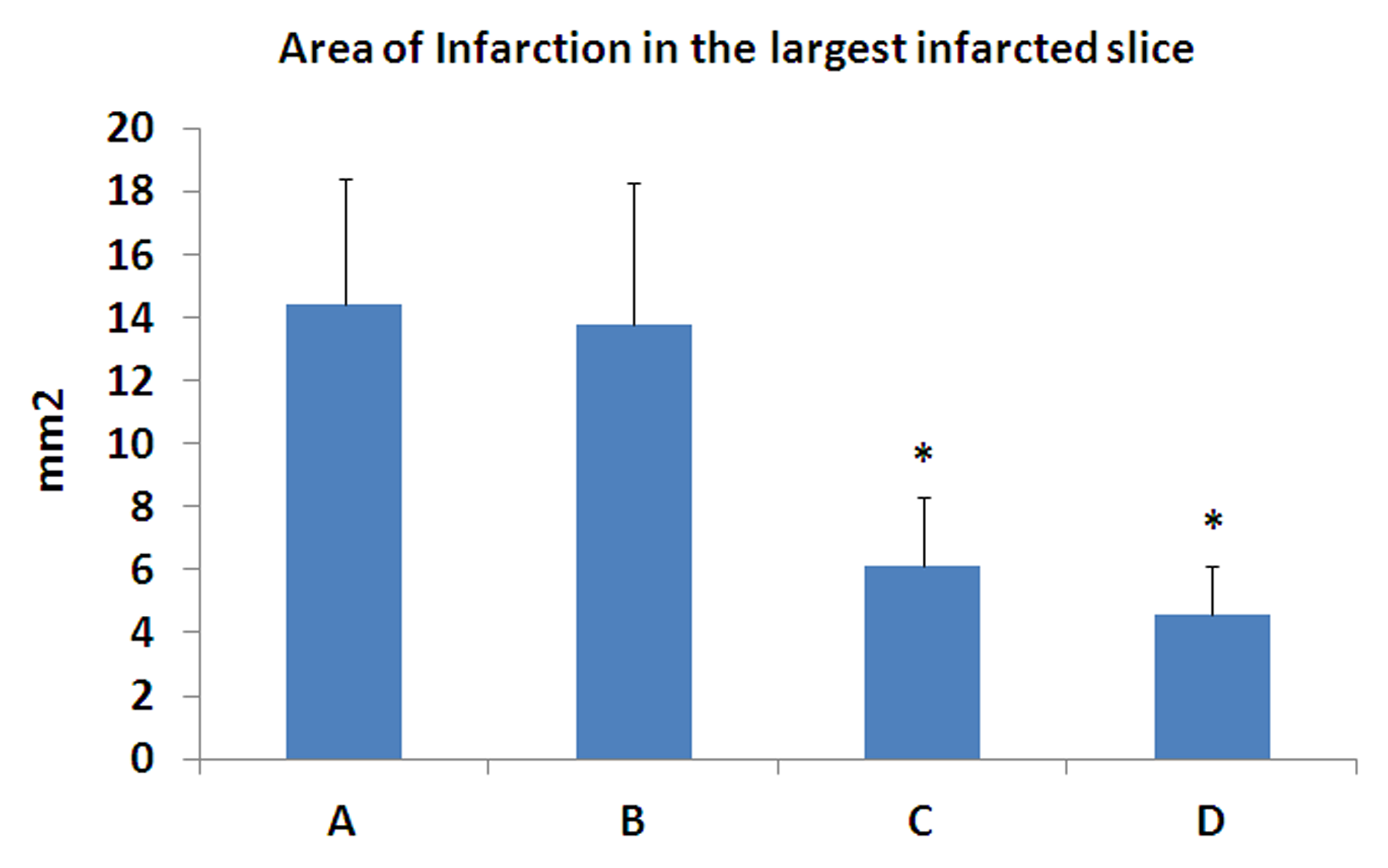

Supplement: Figure S9 — Area of infarction. TTC staining reveals area of infarction (mm2) in the laragest infarcted slice. *p<0.05 versus A or B. (TIF) [file pone.0091408.s009.tif]
